# The Relevance of G-Quadruplexes for DNA Repair

**DOI:** 10.3390/ijms222212599

**Published:** 2021-11-22

**Authors:** Rebecca Linke, Michaela Limmer, Stefan A. Juranek, Annkristin Heine, Katrin Paeschke

**Affiliations:** 1 Department of Oncology, Hematology, Rheumatology and Immune-Oncology, University Hospital Bonn, 53127 Bonn, Germany; Rebecca.Linke@ukbonn.de (R.L.); Michaela.Limmer@ukbonn.de (M.L.); Stefan.Juranek@ukbonn.de (S.A.J.); annkristin.heine@ukb.uni-bonn.de (A.H.); 2 Department of Biochemistry and Pharmacology, Bio21 Molecular Science and Biotechnology Institute, University of Melbourne, Parkville, VIC 3010, Australia

**Keywords:** G-quadruplex, genome instability, homologous recombination, non-homologous end joining, nucleotide excision repair, translesion synthesis

## Abstract

DNA molecules can adopt a variety of alternative structures. Among these structures are G-quadruplex DNA structures (G4s), which support cellular function by affecting transcription, translation, and telomere maintenance. These structures can also induce genome instability by stalling replication, increasing DNA damage, and recombination events. G-quadruplex-driven genome instability is connected to tumorigenesis and other genetic disorders. In recent years, the connection between genome stability, DNA repair and G4 formation was further underlined by the identification of multiple DNA repair proteins and ligands which bind and stabilize said G4 structures to block specific DNA repair pathways. The relevance of G4s for different DNA repair pathways is complex and depends on the repair pathway itself. G4 structures can induce DNA damage and block efficient DNA repair, but they can also support the activity and function of certain repair pathways. In this review, we highlight the roles and consequences of G4 DNA structures for DNA repair initiation, processing, and the efficiency of various DNA repair pathways.

## 1. Introduction

**Figure 1:**
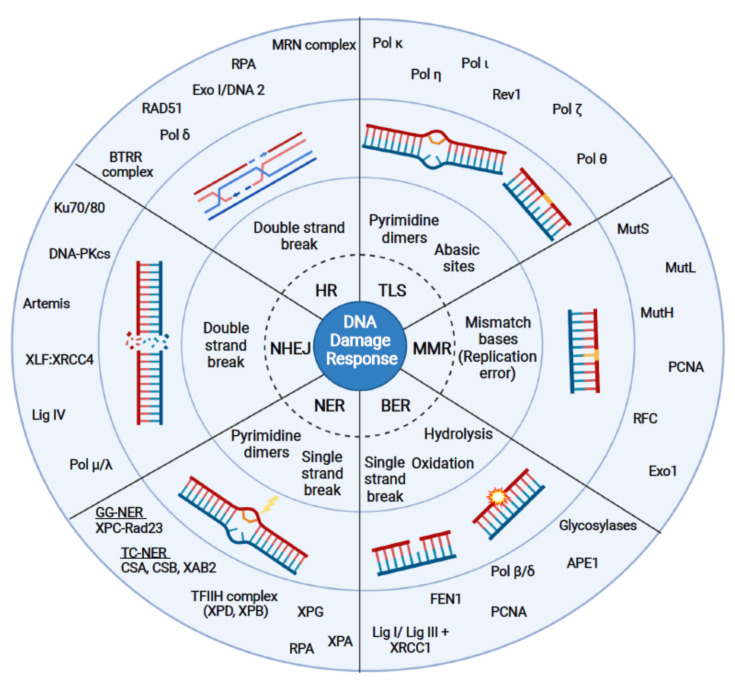
Overview of DNA damage-specific repair pathways. DNA repair mechanisms that are discussed in this review are summarized in this illustration: homologous recombination (HR), non-homologous end joining (NHEJ), base excision repair (BER), nucleotide excision repair (NER), mismatch repair (MMR) and translesion synthesis (TLS). In the inner circle the type of lesion is named and illustrated in the middle circle. In the outer circle major proteins that act during the repair pathway are listed. Figure was created using BioRender.com.

One of the hallmarks of cancer is the loss of genome stability, which is often caused by defects in the DNA damage response (DDR) [1,2,3,4]. Upon DNA damage cells activate an efficient DDR that recognizes and repairs lesions and preserves genome stability [5]. Different types of DNA repair pathways have evolved that differ in their specificity for the type of DNA lesion. There are at least five major DNA repair pathways active in eukaryotes, which function in different cell cycle phases and upon different damage stimuli (Figure 1). Homologous recombination (HR) and non-homologous end joining (NHEJ) are the major repair pathways that detect DNA double- and single-strand breaks, respectively [6,7,8]. After the completion of DNA replication in S phase, different repair pathways are activated in response to DNA damage (post-replicative DNA repair), such as nucleotide excision repair (NER), base excision repair (BER), and mismatch repair (MMR) [9,10,11,12]. Higher eukaryotes have evolved a system known as translesion synthesis (TLS), which supports the bypass of a lesion during DNA replication [13]. If the DNA damage persists, cells can induce cell death (e.g., apoptosis, ferroptosis) [14,15].

DNA damage can be triggered either by exogenous (environmental factors, physical, chemical agents) or endogenous stress [16]. Endogenous DNA damage can arise from reactive oxygen species (ROS), replicative stress as well as the formation of secondary DNA structures. It was demonstrated that the formation of G-quadruplex (G4) DNA structures challenges genome stability [17,18,19,20,21,22]. In a G4 structure guanine–guanine interactions are mediated by the formation of Hoogsteen hydrogen-bonds to form a cyclic arrangement. Such an arrangement is called a G-quartet. Stacking of these G-quartets leads to the three-dimensional structure called G4. G4 structures form within a specific guanine-rich motif harboring four G-tracts that are separated by loop regions (G4 motif) [23]. Based on this consensus motif over 300,000 potential sites are predicted to fold into G4s in humans [24,25]. In vitro analysis, using a polymerase stop assay, demonstrated that in humans 700,000 sites can fold into G4s under specific experimental conditions [26]. Note that not all G4 motifs form at the same time in a cell’s G4 structure, rather a specific subset of G4s that form depending upon cellular conditions [27]. The location of G4 motifs is not random; they are enriched in specific genomic regions such as telomeres, transcriptional start sites and mitotic and meiotic DNA double-strand break (DSB) sites [24,25,28,29]. G4s form in vivo and support cellular pathways such as DNA transcription, translation, and telomere maintenance [30,31,32]. G4 formation and function are mediated by specific proteins that support, recognize, or unfold G4 structures [30].

The regulatory function of G4 structures is contrasted by their potential to induce genome instability. Depending on the location and the time, the formation of G4s can cause genome instability by altering transcription, causing replication fork stalls or inducing mutations [17,21,33,34,35,36,37]. To preserve genome stability helicases, ensure the correct unfolding of G4 structures within cells [38]. In the absence of helicases, such as Pif1 or FANCJ, G4 structures persist and drive genome instability [17,34,37,39,40,41]. The correlation of G4 formation and the formation of DSB is underlined by the finding that regions which are marked by phosphorylation of H2A (γH2A) overlap significantly to potential G4 sites [42,43]. In human cells, damage sites induced by G4-interacting molecule pyridostatin (PDS) correlate with γH2AX sites [44]. It was shown that G4 stabilization/formation by chemical ligands (G4 ligands) can be used as a therapeutic tool to target specific cancer cells [45,46]. Multiple G4 ligands have been developed, which induce G4 formation and slow down the growth of tumor cells [47,48]. Treatment with G4 ligands can induce DNA damage, micronuclei formation, DNA replication pausing, telomere defects and/or transcriptional changes (reviewed in [49]). Although some of these ligands are in clinical trials it is not clear which other cellular pathways they target and why some are specific for only a certain set of cancer entities.

In contrast to their potential to cause DNA damage, G4s can also positively influence certain DNA repair pathways. This observation could allow for a new therapeutic strategy by which G4 structures would be used to support the targeting of DNA lesions and by this enhance genome stability. In other words, can the formation of G4 structures be used as a therapeutic approach to preserve genome stability and enhance DNA repair in diseased cells? To better understand the relevance of G4s for different diseases (e.g., cancer), we summarize recent literature on the impact G4 formation has on the efficiency and function of different DNA repair pathways. We focus on the relevance of G4 formation for the active function of HR, NHEJ, NER, BER, MMR and TLS.

### 1.1. Homologous Recombination (HR)

**Figure 2:**
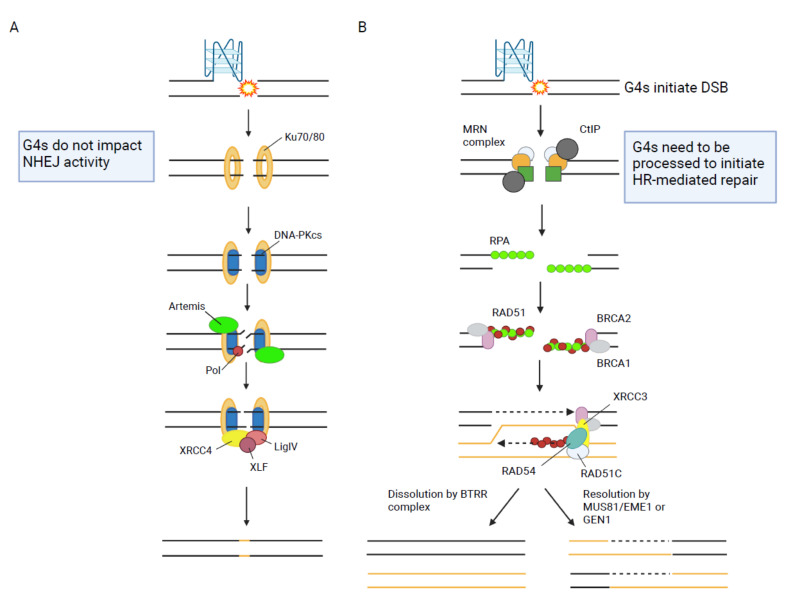
Schematic illustration of canonical DDR. (**A**) Cartoon of essential steps and key proteins during HR. Relevance and consequences of G4 structures are highlighted in the box. (**B**) Cartoon of essential steps and key proteins involved in NHEJ. Relevance and consequences of G4s are highlighted in the box. Figure was created using BioRender.com.

HR is the main pathway used to repair DSB during DNA replication (Figure 1). It is a template-directed repair process that takes advantage of a homologous sequence to ensure the accurate repair of a DNA break [50]. In most cases, the sister chromatid is used as the repair template which restricts HR to the S and G2 phase of the cell cycle when the sister chromatid is present [51]. The mechanism of HR comprises: resection of the DNA break by nucleases and DNA helicases to create single-stranded DNA (ssDNA), invasion of the homologous double-stranded DNA (dsDNA) template, formation of a joint molecule intermediate (single Holliday junction or double Holliday junction), DNA synthesis to restore the missing genetic information and finally the separation of the joined chromosomes by nucleolytic resolution or topological dissolution resulting in cross-over or non-crossover repair products (Figure 2A) [50].

DNA secondary structures, such as G4s, are known barriers for replication fork progression leading to fork stalling or, in more severe cases, fork collapsing followed by DNA DSB [17,18,40,52,53]. G4-mediated DNA damage can lead to micronuclei formation as well as cell cycle checkpoint activation (ATM, ATR) [54,55,56,57]. The HR pathway plays a role in stabilizing stalled replication forks, restarting arrested forks and repairing DSBs arising from collapsing forks. HR represents a mechanism evolved to overcome such replication barriers [58,59,60]. In wild type cells, G4-induced replication stalls and recombination events are prevented by helicases (e.g., Pif1, FANCJ, BLM, WRN) [17,40,41,61,62,63]. In the absence of these helicases, G4 regions can stimulate recombination events and gross chromosomal rearrangement (GCR) [64,65].

The current working hypothesis postulates that G4 structures can lead to replication stalls which are processed by HR to safeguard replication restart and genome stability. Several HR factors have been described in processing or binding to G4s such as MRE11, DNA2, RPA1 and BLM (Table 1) [61,66,67,68]. We will now present the function of these proteins during HR and discuss the relevance of their interaction with G4s for this pathway.

HR is initiated by the MRN complex composed of MRE11, RAD50 and NBS1 that recognizes and binds the DSB and initiates end resection to generate 3′ ssDNA overhangs [103]. Recently, the MRE11 homolog from *S. cerevisiae* and *Oryza sativa* was described to bind to G4 DNA [66,104]. In addition, *S. cerevisiae* Mre11 exhibits endonuclease activity that enables Mre11 to cleave within the G-quartet sites suggesting that Mre11 might provide appropriate DNA termini for replication and repair [66].

End-resection is one of the first steps during HR that is mainly mediated by exonuclease 1 (EXO1) [105]. It has been shown in human cells that EXO1 is important for replication and resection near G4 structures [69]. Besides EXO1, DNA2 functions in the initiation of homology-mediated DSB repair by generating 3′ ssDNA overhangs [106,107,108]. DNA2 function is affected by the formation of G4 structures. DNA binding and functional analysis of helicase activity revealed that yeast and human DNA2 can recognize, bind and unwind intermolecular and intramolecular G4 structures in vitro [67,70]. DNA2-deficiency leads to elevated fragile telomeres, sister telomere associations, telomere loss and the telomere DNA damage response. Telomeric aberrations are significantly increased following treatment with G4-stabilizing molecules [70]. Together, these results suggest that mammalian DNA2 binds and resolves G4 structures to reduce replication stress, support HR and by this promote genome stability. We speculate that DNA2 and/or EXO1 support end-resection by preventing G4 structures.

Later steps during HR are also connected to G4 formation/unfolding. RPA binds and protects ssDNA created during replication and repair from degradation and pairing with the complementary strand [109]. By performing fluorescence resonance energy transfer (FRET), circular dichroism (CD) or electrophoretic mobility shift assay (EMSA) experiments it was shown that human RPA can bind and unfold telomeric and non-telomeric intramolecular G4 structures in vitro [36,68,89,90,91,110,111]. Data from experiments in vivo indicate that RPA prevents G4 formation in particular at telomeres and by this supports DNA replication and repair [112]. During HR, RPA may prevent G4 formation to support strand invasion.

Work in yeast revealed that Rad50 and Rad51 are essential for HR-mediated repair of G4 structures [18]. In addition, work in humans showed that Rad51 as well as BRCA2, key proteins during HR [6], bind and/or modulate G4 structures. Both proteins are required to prevent DNA damage associated with G4 formation [73,75]. In BRCA1-, BRCA2- or Rad51-deficient cells G4 stabilization by G4 ligands (e.g., PDS, 360A) led to increased DNA damage [45,73,75,113,114]. Interestingly, the G4-unwinding helicase Pif1 [62] also participates in G4 unwinding during HR and directly interacts with BRCA2 [115]. One possible model suggests G4 stabilization as an activator of HR, which leads to bypass/repair of G4-mediated DNA damage. Without functional HR, G4 structures accumulate and drive genome instability. Cancer cells lacking a functional HR due to deficiency of BRCA1 and BRCA2 are very sensitive to G4 ligand treatments. In these cancer cells G4 stabilization leads to genome instability and drive cell death.

In the final step of HR, double Holliday junctions (dHJ) are formed with the invading homologous DNA strand during synthesis of the missing genetic information. The dHJ are dissolved by the BLM helicase, which acts along with TOPO3α, RMI1 and RMI2 [116]. Besides its ability to dissolve dHJ, BLM binds and unwinds G4 structures in vitro [61,102]. Sister chromatid exchange events (SCEs) are a byproduct of DSB or collapsed replication forks that are repaired via HR [117,118]. Strand-seq analysis revealed that SCEs are enriched at G4 motifs in BLM-deficient cells indicating that G4 structures can trigger SCE in the absence of BLM [64]. Based on these results it was proposed that failure to unwind G4 structures in BLM-deficient cells leads to replication fork stalling, which triggers recombination, SCE and potentially loss of heterozygosity [64].

Several studies have shown that G4 formation during replication causes replication fork stalling and that G4 structures need to be resolved to initiate HR-mediated repair. We anticipate that the MRN complex, in particular Mre11, senses G4-mediated fork stalling and activates HR.

During HR multiple proteins (e.g., EXO1, DNA2, BLM) prevent the formation of G4s to allow efficient repair of the lesion. However, recombination events are not always disadvantageous for the cells. In specific cells and during specific cell cycle phases programmed and controlled recombination events are beneficial for processes such as meiosis, antigen variation or B-cell development [119,120,121,122]. It has been shown that both antigen variation and class switch recombination (CSR) of immunoglobulins benefit from G4 formation and that similar proteins drive these events [80,123,124,125]. However, details of the relevance of G4 for CSR, meiosis or B-cell development are not fully understood, yet.

### 1.2. Non-Homologous End Joining (NHEJ)

Non-homologous end joining (NHEJ) represents another major pathway for the repair of DSB (Figure 1). NHEJ mediates direct re-ligation of the broken DNA ends [126]. The general mechanism of this pathway comprises DNA end recognition, NHEJ complex assembly and stabilization of the complex at the DSB. After bridging of the DNA ends by the complex, DNA end processing takes place if necessary and the ends areligated (Figure 2B) [7]. It is of great interest whether G4 structures at DSB impact the function of NHEJ proteins. Several proteins involved in the initiation of NHEJ are affected by G4 structures (Table 1) but how this contributes to NHEJ is still unclear.

The NHEJ pathway is initiated by the Ku70/80 heterodimer, which recognizes and binds to the DSB to prevent end resection and serves as a scaffold to recruit other NHEJ components [127]. Besides its role in DNA repair the Ku proteins have been shown to be involved in the maintenance of telomere length and the regulation of transcription [128,129]. During transcription the Ku70/80 heterodimer binds to G4 DNA and RNA structures and alters the transcription of specific genes [76,77]. How and if this is connected to NHEJ has not yet been tested. Furthermore, Ku70 was identified to bind and stabilize the tetrahelical form of the Fragile X syndrome *d(CGG)_n_* expanded sequence [130]. *d(CGG)_8_* can also fold into a G4 [131] but how this is linked to repeat expansion and Ku70 function is not fully understood. The current model is that NHEJ competes with repeat expansion at DSB intermediates [132].

The Ku heterodimer also recruits the DNA-dependent protein kinase catalytic subunit DNA-PKcs to DSB ends. The subsequent phosphorylation of DNA-PKcs is crucial for the induction of NHEJ [133]. In addition to its role in NHEJ activation DNA-PKcs localizes to mammalian telomeres and contributes to telomere length homeostasis and chromosome end protection [134,135,136,137]. Studies in human cells demonstrated a role of DNA-PKcs-dependent NHEJ in the generation of sister telomere fusions as a consequence of G4 formation and/or stabilization by the G4 ligand 360A [73]. It was shown that DNA-PKcs-deficiency exclusively induces chromatid fusions involving leading-strand telomeres. This indicates a role of DNA-PKcs in reestablishing a protective terminal structure specifically on telomeres replicated by leading-strand DNA synthesis [138]. This observation is consistent with a previous study in which DNA-PKcs was demonstrated to be required to refashion the blunt ends of leading-strand telomeres after replication [134]. G-overhangs at lagging-strand telomeres prevent sister telomere fusions. Thus, it was proposed that G4s on the parental telomere “G strand” lead to the resection of the G-overhang at the lagging-strand telomeres. This interferes with DNA-PKcs activity at leading-strand telomeres and subsequently causes NHEJ-mediated sister telomere fusions [73]. Emerging evidence describes G4 stabilization in combination with dysfunctional DNA repair machinery as a promising target for cancer therapy (reviewed in [47]). It was proven that G4 stabilization by pyridostatin (PDS) in a cotreatment with the DNA-PKcs inhibitor NU4771 can induce synthetic lethality in cancer cells [114].

PARP3 (poly (ADP-Ribose) polymerase family member 3) is a protein that interacts with several NHEJ repair factors including DNA-PKcs, Ku70/80 and APLF [139,140]. Studies using PARP3-depleted murine spleen nuclear extracts and mammalian cells containing intrachromosomal NHEJ-reporter substrates have demonstrated that the loss or silencing of PARP3 reduces the efficiency of NHEJ in vitro and in vivo [141]. Furthermore, PARP3-depleted cells are sensitive to PDS, which implies that PARP3 interacts with G4 structures. In immunoprecipitation experiments, it was observed that PARP3-deficient cells exhibit increased G4 levels compared to wild-type cells at sites flanking DSB within G4-rich regions [79]. Based on this data it was suggested that PARP3 negatively regulates G4 structures following DSBs to facilitate repair. In the absence of PARP3 G4 structures accumulate at DSB sites and delay the DNA repair by preventing the deposition of repair factors [79].

NHEJ is also essential for programmed DDR during V(D)J recombination. RAG proteins, which are recombinases that target recombination signal sequences (RSS) within immunoglobulins, can target and cleave non-B-DNA structures such as G4s [142,143]. The protein AID binds to RNA G4s and supports targeting of DNA during V(D)J recombination [80,81]. ChIP-seq analysis links AID-binding to genes that harbor a G4 motif [144]. These investigations are still at their beginning stages, but show the importance of G4 formation for V(D)J recombination. During V(D)J recombination non-canonical DDR (ncDDR) is also activated [145], which is essential to drive gene activation needed for B-cell development. Whether or not these events depend on G4 structures has not been determined yet. In summary, these studies demonstrated that NHEJ efficiency is not affected by G4s. However, in the case of immunoglobulins, G4 structures alter the function of AID and RAG that directly correlate to V(D)J efficiency.

### 1.3. Base Excision Repair (BER)

**Figure 3:**
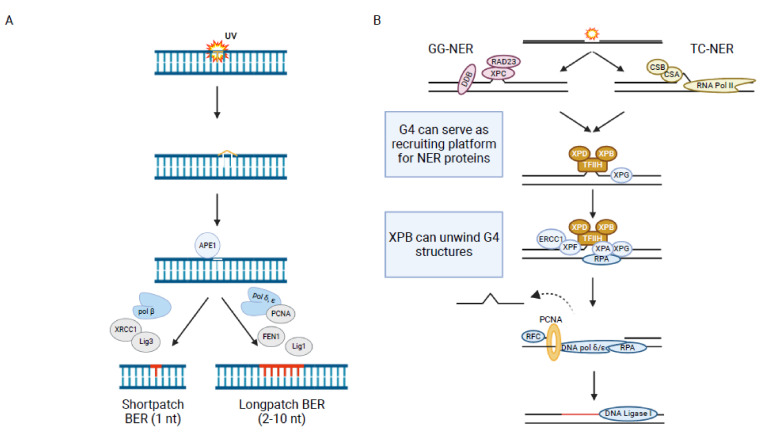

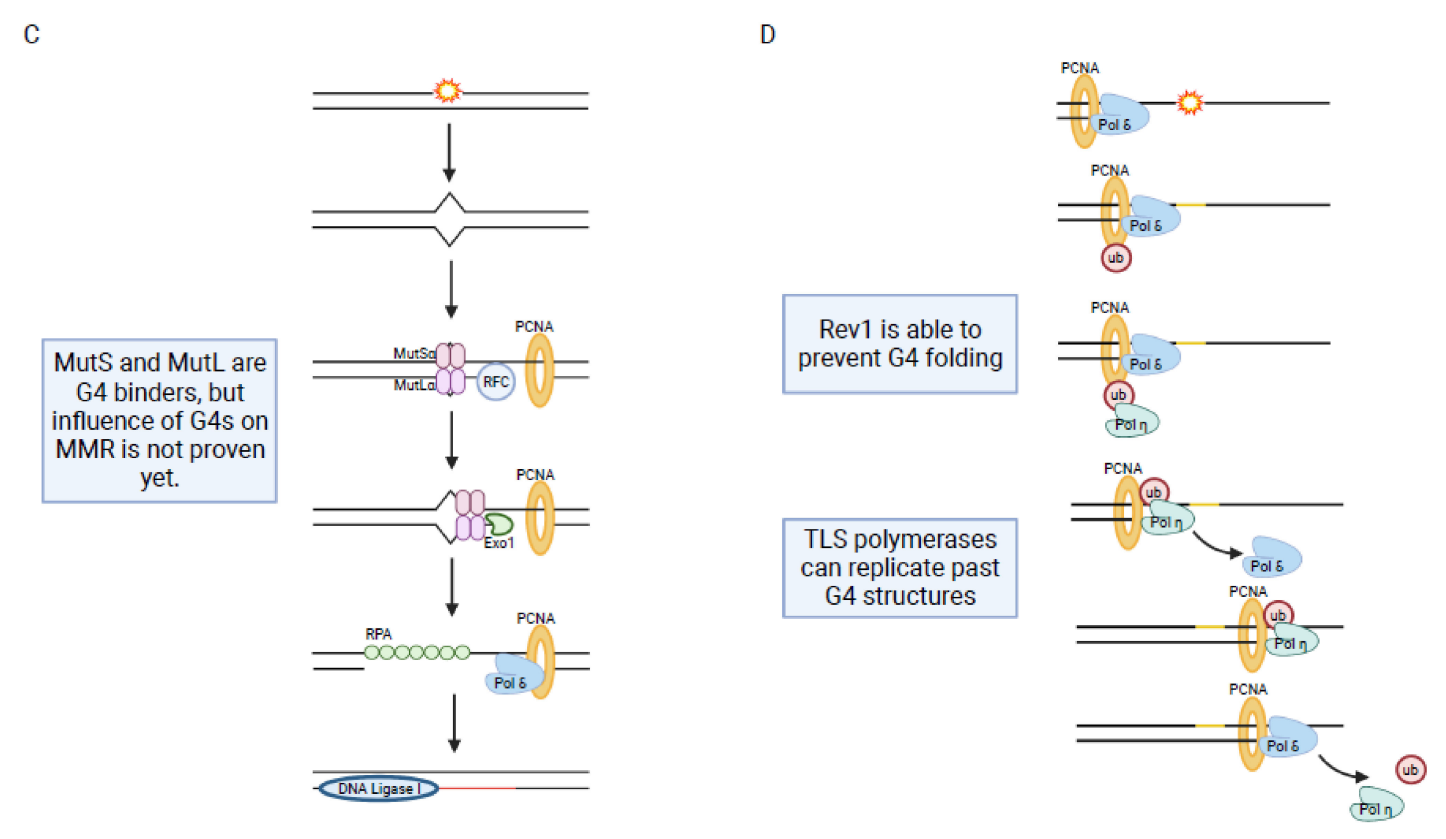
Schematic illustration of post-replicative DDR and translesion synthesis. (**A**) Cartoon of essential steps and proteins during BER. Relevance of G4 formation during initiation and lesion processing are highlighted in the box. (**B**) Cartoon of essential steps and proteins during NER. Relevance of G4 formation is indicated in the box. (**C**) Cartoon of essential steps and proteins during MMR. Relevance of G4 formation is indicated in the box. (**D**) Cartoon of essential steps and proteins during TLS. Relevance of G4 formation during initiation and lesion processing is highlighted in the box. Figure was created using BioRender.com.

The major source of endogenous DNA damage is ROS. They can lead to different DNA base lesions such as thymine glycol (Tg), 7,8-dihydro-8-oxoguanine (8-oxoguanine, oxoG), 4,6-diamino-5-formamidopyrimidine (Fapy-A) or 2,6-diamino-4-hydroxy-5-formamidopyrimidie (Fapy-G) that are repaired by BER (Figure 1) [146,147]. BER is initiated by a DNA glycosylase that removes a large spectrum of alkylated, oxidized or deaminated bases. This process generates an abasic site (apurinic/apyrimidic AP), which iscleaved by the endonuclease APE1 [148]. The resulting single strand break (SSB) is further processed by either of two sub-pathways: “short-patch” BER, a mechanism which replaces only one nucleotide or “long-patch” BER, a mechanism which replaces 2–10 nucleotides (Figure 3A).

Guanines have the lowest redox potential among the four bases and are therefore more susceptible to oxidation [149]. 8-OxoG is the major oxidation product of DNA guanine [150]. Due to their G-rich nature G4 motifs are vulnerable targets for oxidation [151,152,153,154,155]. Regardless of the base substitution after damage within the G4 structure (guanine to 8-oxoguanine, guanine to adenine substitutions or guanine abasic lesion) the same model is followed. The type of lesion is not important, but rather the position of the lesion determines the impact on the stability and conformation of the G4 structure [151,153,154,155]. A conformational change of the G4 structure can lead to gene expression changes, telomerase activity and/or BER initiation [156].

In humans 11 different lesion-specific DNA glycosylases have been identified, which can be classified into four structural families [157]. The H2tH glycosylase family (NEIL1-3) has an RG-rich domain which is known to bind to G4 structures [158]. Glycosylase activity on ROS-damaged telomeric G4 sequences revealed that some glycosylases are not able to remove 8-oxoG from G4 structures. An example for this is 8-oxoG glycosylase 1 (OGG1), the major enzyme for 8-oxoG base damage [159]. However, other oxidized lesions at telomeric G4s (Tg, guanidinohydantoin (Gh) and spiroiminodihydantoin (Sp)) can be removed by the structural families H2tH (NEIL1, NEIL2, and mNeil3) and HhH-GPD (OGG1 and NTH1) [152,159,160]. Recent studies showed that cleavage of lesions within G4s is mainly achieved by members of the H2tH structural family and that the position of the lesion determines if glycosylases are active at these lesions [161]. Similarly, the activity of uracil glycosylases is also affected by the position of the lesion within the G4s. It was shown that uracil excision by bacterial and human uracil glycosylases was significantly reduced at uracil bases positioned directly 5′ or 3′ of G4-tracts [162]. Upon moving the uracil three nucleotides away from the G4-tracts nearly full glycosylase activity was restored demonstrating that the position of the uracil relative to the G4 structure affects the cleavage efficiency. This data suggests that the topology of the G4 structures (e.g., parallel, anti-parallel, hybrid) as well as the position of the lesion changes the activity of glycosylases on the oxidized G4s [152].

APE1 is the endonuclease utilized during BER. After OGG1 generates an AP site [163] APE1 binds to this region [164,165]. Genome-wide mapping of APE1- and OGG1-binding sites revealed a significant overlap to G4 motifs [84]. Loss of OGG1 or APE1 leads to reduced G4 structures, resulting in changes in G4-mediated gene expression. This can be explained by the finding that APE1 promotes G4 formation which facilitates the binding of transcription factors that alter gene expression such as MAZ that alter gene expression [84]. Acetylated APE1 is able to block the activity of WRN [85], a G4 unwinding RecQ helicase, which further stabilizes G4 structures. Deacetylation of APE1 by SIRT1 leads to the dissociation of APE1 from G4 structures [84].

G4s are vulnerable to oxidative stress and are overrepresented in promoter regions. Upon oxidative stress the expression of genes that have G4 motifs in their promoter region changes (e.g., oncogene and proinflammatory genes) [166,167,168]. Expression of DNA repair proteins needs to be upregulated in response to oxidative stress. With this in mind, a model was proposed in which G4s act as a sensor for oxidative damage and support gene expression to modulate cell proliferation, the innate immune response and BER activity [163,169,170,171]. However, are G4 motifs in genes relevant for BER and do they support the activation of BER after oxidative damage? The DNA glycosylases NEIL3, NTHL1 (endonuclease III-like protein 1) and PCNA (proliferating cell nuclear antigen) harbor G4 motifs within the coding-strand of their promoter [172]. These regions are prone to accumulating oxidative stress. Interestingly, they all have five runs of guanines within the G-tracts of their G4 motif. To form a G4 structure only four G-tracts are required. The fifth acts as a “spare tire” which allows G4 formation even if one G-tract is blocked [163,171,173,174]. This data led to a feedback loop model in which DNA lesions at repair genes drive BER and support genome stability during oxidative stress [175].

In summary, oxidation within G4s can lead to a conformational change which is targeted by glycosylases but can also reduce glycosylase activity. OGG1 cleaves the 8-oxoG and creates an AP site. The AP site changes the thermal stability of the duplex which allows the duplex to open. This opening together with APE1-binding supports G4 formation. The AP site is in the loop region of the G4 and bound by APE1 [176]. After acetylation of APE1 (AcAPE1), the G4-AcAPE1 complex drives G4-mediated transcriptional changes. The binding of transcription factors is also linked to the presence of APE1 (e.g., STAT3, HIF-1a, NF-kB, HDAC1) [171,177,178,179].

G4 structures have a dual role during BER. They modulate glycosylase binding and activity but also promote BER as G4 formation supports the expression of BER genes.

### 1.4. Nucleotide Excision Repair (NER)

Nucleotide excision repair (NER) is the canonical DNA repair pathway that is activated upon ultraviolet light (UV) irradiation or by bulky chemical compounds which lead to the formation of thymine dimers or T-C (6–4) photo products (Figure 1). In eukaryotes NER is divided into two sub-pathways: global-genome NER (GG-NER) and transcription-coupled NER (TC-NER). GG-NER takes place anywhere in the genome while TC-NER is restricted to regions of active transcription. They also differ in the initiation of the repair. In GG-NER the damage is recognized by XPC-RAD23 [180] while in TC-NER the proteins CSA, CSB and XAB2 are responsible for detecting damage-mediated stalling of RNA Pol2 [181]. Due to the dense chromatin structure a protein complex called UV-DDB helps to relax the chromatin to facilitate the binding of XPC [182]. After recognition of the damaged DNA site, the repair process continues similarly in both pathways. First, the endonucleases XPG, XPA, RPA and the TFIIH complex bind to the DNA damage site. The TFIIH complex makes a first cut 5′ to the damage site. Two subunits of TFIIH, the helicases XPD and XPB, unwind the DNA double strand. XPG generates a second cut 3′ to the initial damage site. XPA and RPA facilitate the removal of a 25–30 oligodeoxynucleotide generating a gap around the damaged site. The gap is subsequently filled by members of the DNA replication machinery (Figure 3B) [183]. Defects in NER are linked to several inherited genetic diseases such as Xeroderma pigmentosum, which is connected to a high risk of skin cancer development [184].

Recently, much data have been collected, demonstrating how G4 structures impact NER. Work in yeast positively correlated G4 formation with the initiation of NER after UV damage [86]. Using the G4-specific antibody BG4, a two-fold increase of G4 structures was detected after UV irradiation. These G4 structures were bound and stabilized by the protein Zuo1. Zuo1 is a DNAJ protein, which is present in the cytoplasm and nucleus. It has diverse functions within the cell [185,186]. Using ChIP-qPCR it was demonstrated that Zuo1 stabilization of G4 structures supports the recruitment of NER proteins like Rad4 or Rad23 (homolog of XPC and RAD23). Without Zuo1 less G4s form and cells become UV sensitive. This work demonstrates how G4 formation and stabilization positively influences the initiation and repair of UV lesions [86]. The human orthologue of Zuo1, ZRF1, was found to support the NER recruitment via interaction with the UV-DDB complex. If and how G4s are linked to ZRF1 function during NER is not clear, yet. Mms1, which mimics the yeast ortholog of DDB1 (part of the UV-DDB complex), also binds to G4 structures [187,188]. This led to the speculation that DDB complex also plays a role in G4-mediated DNA repair in connection with ZRF1.

In addition to the initiation of NER later steps of NER are also affected by G4 structure formation. ChIP-seq in human cells demonstrated that binding sites of XPB and XPD overlap with G4 motifs [88]. Analyses in vitro showed that the well-conserved archaea homolog of XPD and XPB binds robustly to G4s [88]. XPD exhibits robust helicase activity on G4 structures in the presence of ATP whereas XPB shows no helicase activity at G4 structures [88]. One hypothesis on why XPB and XPD bind to G4 structures is that they coordinate and support NER but also DNA replication and transcription within the TFIIH complex. Especially the binding of XPB is enriched at promoters that are activated upon UV damage. This indicates a potential role of XP-family members in controlling gene expression. How or if this is connected to G4 structures is not clear but opens the possibility that G4-driven gene expression changes might support NER function.

Polymerase κ (Pol κ), which is known to replicate past G4 structures in the TLS pathway (see TLS section), may also be connected to NER. The depletion of Pol κ in mouse cells showed a decrease in NER activity [189]. We speculate that Pol κ may support replication past G4 sites during NER by replacing or aiding replicating polymerases.

### 1.5. Mismatch Repair (MMR)

Mismatch repair (MMR) is a post replicative DNA repair mechanism, which is responsible for correcting spontaneous base-base mispairing and small insertion-deletion loops (Figure 1). MMR was initially identified in *E. coli* but most proteins are highly conserved in humans [190]. In humans, MMR mostly acts at actively transcribed genes [191]. The proteins involved in MMR occur as heterodimers. MSH2-MSH6 (MutSα) can sense single base mismatches while MSH2-MSH3 (MutSβ) can detect larger insertion-deletion loops (around 13 nucleotides) [192]. The DNA-bound heterodimer recruits MLH1/PMS2 (MutLα) as well as PCNA, replication factor C (RFC) and exonuclease 1 (Exo1). The damaged DNA isdegraded and DNA synthesis restarted [12] (Figure 3C). Mutation in genes of the MMR proteins can lead to cancer, for example endometrial and colorectal cancer [193,194].

In addition to the previously described DNA repair pathways, proteins of the MMR are also linked to G4 structure formation. A direct contribution of G4 structures to MMR activity has not been observed, yet. The protein for the initiation of MMR, MutS (*E. coli*), binds to G4 structures with a higher affinity than to G/T mismatches in vitro [93,94]. A replacement of phenylalanine at position 36 in MutS, which is necessary for heteroduplex recognition and mismatch correction, results in the loss of binding to G/T mismatches although the binding to G4 structures remains [94]. This indicates that binding of MMR proteins to G4s is independent of MMR activation.

MutL has a coordinative and endonucleolytic function during MMR [195,196,197]. MutL binds robustly to parallel G4 structures. G4 structures do not affect the MMR because they are not recognized as a DNA damage site. This is supported by the finding that activation of MMR is not inhibited when there is a G4 structure and a G/T structure at least 17 bp apart [93]. This raises the question why MMR proteins recognize G4 structures: does binding to G4 structures stimulate gene expression of proteins essential for MMR (similar to BER) or is G4 structure formation a positive signal that supports MMR like it does during NER.

### 1.6. Translesion Synthesis (TLS)

Translesion synthesis (TLS) is known as a damage tolerance repair pathway that acts at lesions that cause replication pausing such as bulky adducts, apurinic sites, UV-mediated pyrimidine dimers, G4 structures or hydrolyzed bases (Figure 1) [198]. During TLS the damage is bypassed by the action of specialized polymerases that use the DNA lesion as a binding template and mediate the bypass of the lesion. DNA-polymerases from the Y family (polymerase η, ι, κ and REV1) and from the B family (polymerase ζ) act during TLS. In general, the DNA lesion is not repaired during TLS [199,200].

Conventional DNA replication polymerases such as Pol α, Pol δ and Pol ε stall at G4 structures, which can lead to genome instability [198,201,202,203,204]. G4 structures can impair DNA replication causing mitotically inheritable replication blocks which cause genome instability [205]. In detail, replication stalls at the G4 structure and leaves strand gaps behind, which will cause the formation of a DSB in the next cell cycle phase [205]. This G4-dependent replication stalling can be prevented by the action of G4-unwinding helicases during DNA replication (28). Many different helicases have been identified that show robust G4 unwinding activity (Pif1, WRN, BLM, FANCJ) and support genome stability (Figure 3D) [38].

Replication through G4 structures via TLS has been investigated in multiple studies. The polymerases REV1, eta (Pol η), kappa (Pol κ), theta (Pol θ) and potentially zeta (Pol ζ) can act on G4 structures during TLS [206]. REV1 can bind directly to G4 structures where it may support G4 destabilization [97]. REV1 influences TLS at G4 structures in two manners: first, by coordinating the interaction of PCNA, ubiquitin and polymerases ε and κ during TLS [199,207,208] and second, by using its catalytic activity as a deoxy-cytidyl transferase on the G4 [209,210]. REV1 is a specialized polymerase that incorporates only cytosines. The hypothesis is that REV1-binding competes with G4 structure formation. REV1 is incorporating cytosines opposite of the first guanines of the G-tract, which may prevent G4 folding. This process could be supported by the binding of Pol κ and Pol ζ [98].

After replication, nucleosomes need to reassemble at the nascent DNA. Histone recycling and re-establishment can be disturbed due to replicative stress [211]. G4 formation can contribute to epigenetic deregulation after DNA replication, which is linked to REV1 (but also WRN, BLM) function at G4s. In REV1-deficient cells changes in histone modifications were detected in the vicinity of G4 motifs that drive transcriptional changes [19,212,213]. Whether or not this is linked to REV1 function during TLS, and following changes in gene expression support, TLS has not been investigated to date.

Pol η and Pol κ are known to support replication past G4 sites [96]. Pol η was shown to have more than 25% more activity at the G4 structure with 100-fold more accuracy in copying G4 sites than the replication polymerase Pol ε [214]. Human Pol κ has a decreased fidelity and an enhanced activity two nucleotides before the G4 motif, which may suggest that Pol κ prepares for synthesis before the G4 motif [203]. Even though both polymerases can support replication past G4 structures, G4s are a stronger hindrance for Pol κ than for Pol η [203]. Pol κ most likely also acts on other repetitive DNA structures because downregulation of Pol κ leads to DNA break formation at such sites [96]. In summary, TLS polymerases are activated in case DNA replication stalls at G4 structures. This is initiated by a fast switch between replicative polymerases to TLS polymerases.

TLS is very error prone. It has been suggested that polymerase theta (Pol θ) functions at G4 structures to prevent mutagenesis as the last instance of TLS [215]. In *Caenorhabditis elegans* DSBs caused by fork stalling at G4 sites are repaired via Pol θ-mediated end joining (TMEJ) [99]. Deletion of Pol θ causes large deletions around the G4 structures which are even more severe in the absence of the FANCJ helicase dog-1 [21]. This indicates that Pol θ prevents large scale deletions and subsequent genome instability at G4 structures. In summary, TLS polymerases are needed to prevent G4-mediated genome instability by replicating past them, while REV1 even seems to prevent folding of the G4 structure.

It has been demonstrated that G4 structures are cellular tools that actively form to support cellular functions such as transcription, telomere maintenance (reviewed in [30]) and some steps during BER or NER (see above). As TLS does not remove or alter G4 structures it is likely that specific proteins bind to G4 structures to prevent TLS. In yeast it has been shown that Zuo1 binds to G4 structures and recruits the NER machinery. In addition, Zuo1 binding to G4 also prevents the binding of Rev1 [86]. These findings show that G4 formation can support the activation of a specific DNA repair pathway but also prevent the binding of an alternative repair mechanism. Whether this process only occurs in yeast has not yet been investigated. However, the sum of G4 interacting proteins that are linked to DNA repair suggests that G4 function and relevance for DNA repair pathway activation is more complex than current models describe.

## 2. Conclusions

**Figure 4:**
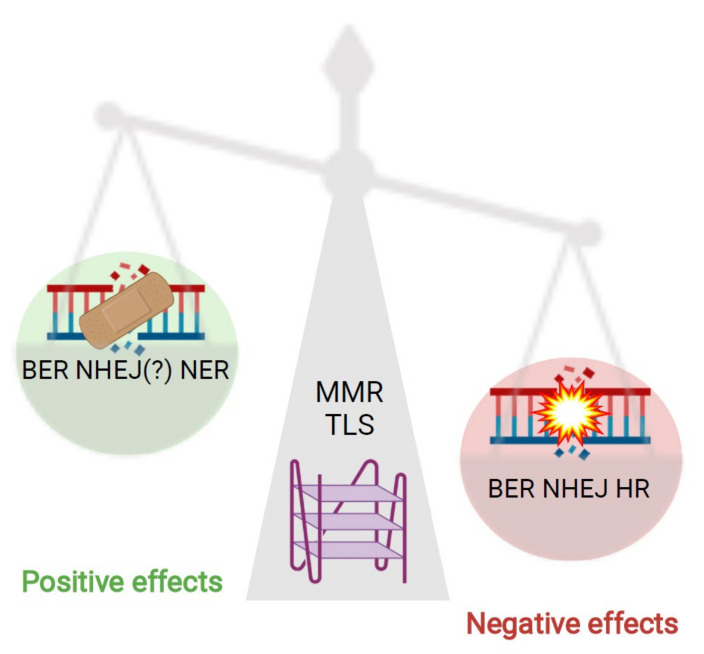
Impact of G4s on DNA repair pathways. G4 DNA structures can influence the function of DNA repair mechanisms but can also contribute to genome stability. On the one hand G4 structures positively affect the repair of DNA damages recognized and repaired by NER. On the other hand, G4 structures have a negative impact on the repair efficiency of HR and NHEJ and MMR. BER can be both positively and negatively affected by G4 structures. TLS is not affected by G4 structures as it is able to replicate through these secondary structures to maintain genomic stability. Figure was created using BioRender.com.

In this review, we presented and discussed the relevance of G4 structures for the function and efficiency of DNA repair pathways. The findings summarized in this review reveal that G4 structures impact genome stability by modulating different aspects of DNA repair (Figure 4). Multiple proteins involved in DNA repair can bind to or interact with G4 structures (Table 1), highlighting the importance and relevance of these structures for DNA repair pathways. Note that in the table we have summarized all proteins relevant for DNA repair that have been shown to bind to or interact with G4 structures. Those marked with an asterisk were identified by different screening methods but have not yet been experimentally validated or characterized. Once these are further characterized (and more potential proteins are described) we will better understand how and when G4 structures positively or negatively modulate DNA repair efficiency. G4 structure formation and stabilization is currently investigated as a potential anti-cancer drug that enhances genome instability and modulates transcription and telomerase homeostasis. There are new therapeutic approaches that combine G4 structure stabilization and the blocking of an efficient DNA repair pathway (reviewed in [47]). A detailed understanding about the relevance and consequences of G4 structure formation for DNA repair activation but also the modulation of its efficiency will further stimulate the utilization of G4 structures as an anti-cancer tool.

## Figures and Tables

**Table 1:** In this table proteins from different DNA damage pathways, which are functioning at G4s, like binding, unwinding, stabilizing, and so forth, are summarized. The organism in which the G4 interaction was shown is listed. The function which is marked with an asterisk (*) is not proven, yet.

Pathway	Protein	Function at G4s	Organism	REFs
HR	EXO1	Binding and unwinding *	Human	[69]
	DNA2	Binding and unwinding	Human/yeast	[67,70]
	Rad50	Binding *	Human	[71,72]
	Rad51	Necessary for G4-mediated DNA damage	Human	[73]
	BRCA1	Binding	Human	[74]
	BRCA2	Necessary for G4-mediated DNA damage	Human	[75]
	Mre11	Binding	Yeast	[66,71]
NHEJ	Ku70	Binding	Human	[76,77,78]
	Ku80	Binding	Human	[76,77]
	PARP3	Affecting G4 levels	Human	[79]
	AID	Binding	Human	[80,81,82]
	PARP1	Binding	Human	[78,83]
BER	OGG1	Supporting formation	Human	[84]
	APE1	Supporting formation	Human	[84,85]
	PARP1	Binding	Human	[78,83]
NER	Rad23	Binding *	Yeast	[86]
	CSB	Binding and resolving	Human	[87]
	XAB2	Binding *	Human	[71]
	XPD	Binding and unwinding	Human	[88]
	XPB	Binding	Human	[88]
	RPA	Binding, preventing formation, unfolding	Human	[68,89,90,91,92]
	DDB2	Binding *	Human	[78]
	Zuo1	Binding and stabilizing	Yeast	[86]
MMR	MutS	Binding	Bacteria	[93,94]
	MutL	Binding	Bacteria	[93]
	MSH2	Binding	Human	[95]
	MSH6	Binding	Human	[95]
	MSH4	Binding *	Human	[78]
	Exo1	Binding and unwinding *	Human	[69]
TLS	Pol η	Replicating past G4s	Human	[96]
	Pol κ	Replicating past G4s	Human	[96]
	REV1	Binding, preventing refolding	Human	[97,98]
	Pol θ	Preventing deletions at G4s	Human	[99]
Helicases	WRN	Unwinding	Human	[71,100,101]
	FANCJ	Unwinding	Human	[39]
	BLM	Binding and unwinding	Human	[61,102]
	Pif1	Binding and unwinding	Yeast	[17,40]
